# Strategies to Improve Care in the Emergency Department for Culturally and Linguistically Diverse Adults: a Systematic Review

**DOI:** 10.1007/s40615-023-01876-z

**Published:** 2023-12-20

**Authors:** Nematullah Hayba, Colleen Cheek, Elizabeth Austin, Luke Testa, Lieke Richardson, Mariam Safi, Natália Ransolin, Ann Carrigan, Reema Harrison, Emilie Francis-Auton, Robyn Clay-Williams

**Affiliations:** 1 Australian Institute of Health Innovation, Macquarie University, Level 6, 75 Talavera Road, North Ryde, 2109 Australia; 2 Internal Medicine Research Unit, Department of Regional Health Research, University Hospital of Southern Denmark, Aabenraa, University of Southern Denmark, Odense, Denmark; 3 Construction Management and Infrastructure Post-Graduation Program (PPGCI), Federal University of Rio Grande Do Sul (UFRGS), Porto Alegre, Brazil

**Keywords:** Disparity, Health equity, Ethnic or racial minority, Discrimination, Social accountability, Humility

## Abstract

**Background:**

The emergency department (ED) is an important gateway into the health system for people from culturally and linguistically diverse (CALD) backgrounds; their experience in the ED is likely to impact the way they access care in the future. Our review aimed to describe interventions used to improve ED health care delivery for adults from a CALD background.

**Methods:**

An electronic search of four databases was conducted to identify empirical studies that reported interventions with a primary focus of improving ED care for CALD adults (aged ≥ 18 years), with measures relating to ED system performance, patient outcomes, patient experience, or staff experience. Studies published from inception to November 2022 were included. We excluded non-empirical studies, studies where an intervention was not provided in ED, papers where the full text was unavailable, or papers published in a language other than English. The intervention strategies were categorised thematically, and measures were tabulated.

**Results:**

Following the screening of 3654 abstracts, 89 articles underwent full text review; 16 articles met the inclusion criteria. Four clear strategies for targeting action tailored to the CALD population of interest were identified: improving self-management of health issues, improving communication between patients and providers, adhering to good clinical practice, and building health workforce capacity.

**Conclusions:**

The four strategies identified provide a useful framework for targeted action tailored to the population and outcome of interest. These detailed examples show how intervention design must consider intersecting socio-economic barriers, so as not to perpetuate existing disparity.

**Registration:**

PROSPERO registration number: CRD42022379584.

## Background

Emergency departments (EDs) seek to provide equitable health access to all. Equitable health access has been defined as having access to appropriate information; having access to services that are relevant, timely, and sensitive to the person’s needs; and being able to use the health service with ease, having confidence that you will be treated with respect [1]. Pursuing health equity, however, means healthcare services must be sensitive to, and seek to overcome, existing social inequalities that individuals experience by virtue of their belonging to particular social, religious, political, ethnic, or occupational groups [2, 3]. Discriminatory policies, lack of available services in rural areas, poor housing, pay gaps, limited access to education, lower health literacy, lower socioeconomic status, a lack of social capital, and language barriers are examples of often intersecting structural and social determinants that contribute to inequitable health access and poorer health outcomes. Inequities are referred to as a subset of inequalities that are deemed unfair as they may be avoidable [2]. Healthcare services that overlook social and cultural considerations when delivering care may perpetuate existing inequities in health outcomes [3, 4], leading to health disparity. There are known health inequities and disparities in health outcomes for patients who are culturally and/or linguistically diverse (CALD).

The term ‘CALD’ is used in Australia as a multidimensional approach to understanding the diverse individuals and communities distinguishable from the majority population through one or more characteristics such as cultural tradition, a common geographic origin, language, or religion [5]. Internationally, the term ‘ethnic minorities’ is also commonly used to describe these groups. Population diversity is an increasing feature of demographic change, particularly with large numbers of people being displaced through conflict or through greater immigration. The ten countries that experienced the most migration in 2020 were the United States of America (USA), Germany, Saudi Arabia, Russia, the UK, United Arab Emirates, France, Canada, Australia, and Italy [6, 7]. In 2020, there was an estimated 281 million international migrants in the world, equating to 3.6% of the world’s population [7]. For recent migrants in particular, the ED is an important entry point into the healthcare system. An individual’s experience of the health system impacts the way they seek care in the future.

Compared to the population majority, individuals from a CALD background present later to ED, with more severe illness [8], and re-present at higher rates [9]. These behaviours are thought to reflect the social vulnerability and reluctance or difficulty in accessing primary care for these patients [10, 11]. Patients have also reported racial discrimination and expressed distrust of clinicians [4, 12, 13], which may be associated with delay in seeking healthcare, or non-adherence to prescribed treatment [13]. For some, the ED may act as a ‘safety net’, providing critical healthcare access irrespective of pay or insurance coverage [10]. In the ED, there may be a lack of ethnicity-specific care such as spoken language assistance [14]. Analyses show CALD patients spend significantly longer than others in the ED [15], with their length of stay heightened by communication difficulties [16–18]. Healthcare providers, researchers, and consumers have identified institutional and interpersonal factors in health care delivery that perpetuate poorer outcomes for people from a CALD background.

In the USA, people from CALD communities have been found to receive incongruent treatment in ED for common symptoms such as chest pain and acute coronary events, trauma, stroke, and brain injuries and pain management for fractures, migraines, and back pain [16]. Outside the USA, collection of ethnicity data in the ED is less consistent, but there are documented differences from population majorities in provision of analgesia, triage scores, diagnostics, and treatments [19]. A lack of cultural responsiveness among staff, challenges with language, misinterpretation of behaviour, gender dynamics, and inherent stereotypes hinder optimal patient care [6, 20, 21]. In high-pressure and often crowded settings such as the ED, there may be insufficient resources (human and technical) to meet cultural competence [22–26]. Commitment to equity in healthcare for people from a CALD background requires investment, structural change, and interventions that better meet their needs [4, 20].

While recommendations for policies and structures to address disparity for CALD communities are emerging [4, 13, 20], an overview of interventions that have been implemented to improve healthcare for CALD patients in ED is required to guide informed action. We sought to identify interventions implemented in the ED as a foundation for a comprehensive program to codesign new or adapted models of ED care for this cohort [27]. This systematic review aimed to examine the strategies that have been used to improve health care delivery in ED for adults from a CALD background that report measures of patient satisfaction, patient outcomes, staff experience, or system performance.

## Methods

This systematic review adheres to the Preferred Reporting Items for Systematic Reviews and Meta-Analyses (PRISMA) statement [28] and was registered with PROSPERO (registration number: CRD42022379584).

### Search Terms

A systematic literature search was conducted using four health sciences databases: CINAHL, Embase, Medline, and Scopus. Studies published from inception to November 2022 were considered for inclusion. Electronic database searches were supplemented by snowballing for additional articles based on the reference lists of the studies retrieved from database searches. The applied search terms were broad, including synonyms, truncations, and combinations of ‘ethnic minority’, ‘emergency department’, ‘quality improvement’, and ‘patient outcomes’ to ensure all studies that may align with the population, intervention, and outcome criteria were captured. A complete search strategy applied in the electronic database Medline is presented in Supplement 1.

### Eligibility

We included empirical studies that reported implementation and outcomes of interventions or strategies with a primary focus on improving ED care delivery for adults (aged ≥ 18 years) from CALD backgrounds. As this review was undertaken in Australia, we used the Australian definition of CALD, which does not include Indigenous populations [5]. Indigenous Australians are categorised as a specific population group to facilitate recognition of their status as the original and ongoing custodians of the land, and to more effectively target affirmative action to help overcome the significant negative impact that colonisation has had on their health and wellbeing. As not all countries make this distinction, we have included results reported for international studies where Indigenous Peoples are included as an ethnic minority group. We excluded non-empirical studies (e.g. reviews, perspectives, and grey literature), studies where an intervention did not occur in ED, papers where full text was unavailable, and papers published in a language other than English.

**Fig. 1:**
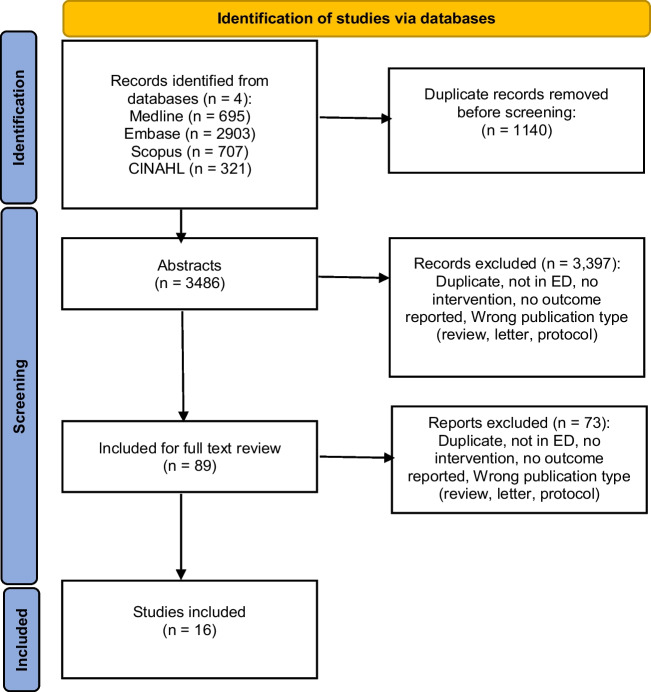
PRISMA diagram [28] of systematic search showing 3486 abstracts reduced to 89 for full-text review based on eligibility criteria and further reduced to 16 included studies

Following removal of duplicates, a random sample of 50 abstracts was screened by all reviewers to ensure concordance and reliability of applying the inclusion/exclusion criteria. The remaining 2268 abstracts were then independently screened by reviewers in pairs, with discrepancies resolved via discussion. The inclusion criteria were then independently applied for full text review of the 89 included papers by seven pairs of reviewers (NH, LB; NH, AC; NH, EA; NH, CC; AC, EA; CC, LB; and RCW, RH). A senior reviewer (RCW) provided final judgement on inclusion for 16 full texts, following discussion (Fig. 1).

### Quality Assessment

The methodological quality of the included peer-reviewed studies was assessed using the following The Joanna Briggs Institute critical appraisal tools: checklist for randomized controlled trials (RCT), checklist for cohort studies, checklist for qualitative studies, and checklist for quasi-experimental studies. The tools selected were based on study design and applied independently by pairs of reviewers (EA, CC, LT, MS, NR), with disagreements resolved via discussion; outcomes of the quality assessment for each paper are provided in Supplement 2. The level of evidence reported was graded using the Australian National Health and Medical Research Council appraisal tool [29]. The ProgressPLUS appraisal [30] was applied to consider socially stratified factors to illuminate inequities; outcomes of the appraisal are provided in Supplement 3.

### Synthesis

All included papers were read in full by two authors (CC, LT), paying attention to the intervention aim, the context, and to whom the intervention was delivered.

CC, LT, and LR discussed the papers and based on domain knowledge and iterative sorting, derived four categories:

1. Patient self-management of health issues — interventions delivered to the patient to assist them to better manage their own health.
2. Communication between patients and the health provider — interventions implemented to overcome existing communication difficulties between patient and provider.
3. Provider adherence to good clinical practice — interventions to improve provider delivery of care.
4. Health workforce capacity — interventions to improve the cultural competence (organisational fluency with cultural diversity through elevating staff knowledge and behaviours) or cultural humility (implicit self-awareness and self-reflection that cultivates respectful interpersonal interactions that are sensitive to power and value differentials) of the provider workforce [31].

As the outcome measures and population groups were not homogenous, reported results were tabulated as reported.

## Results

Sixteen papers met inclusion criteria. The characteristics of the included studies are described in Table 1. Of the 16, 11 (69%) were published in the last 10 years. Thirteen studies (81%) were undertaken in USA and one in each of Australia, Canada, and Israel. We extracted the ethnic groups as defined by the authors: ‘African American’ (*n* = 3); ‘African American, Hispanic’ (*n* = 2); ‘Spanish speakers’ (*n* = 2); ‘Black and ‘Other’ — including Asian, Hispanic/Latino, American Indian, Alaska Native, Pacific Islander, Middle Eastern’ (*n* = 2); ‘Hispanic or Spanish speakers’ (*n* = 1); ‘Mexican-origin Hispanic’ (*n* = 1); ‘Hispanic/mixed origin’ (*n* = 1); ‘Spanish, Mandarin, or Cantonese-speaking’ (*n* = 1); ‘Mandarin, Cantonese, Punjabi, Russian, American Sign Language, and Turkish’ (*n* = 1); ‘Afghanistan, Sri Lankan, Iran’ (*n* = 1); and ‘Arab and non-Jew’ (*n* = 1) (Table 1).

**Table 1:** Characteristics of included studies

Author, year, country	Study type	CALD definition	Strategy type	Study aim	Participants (*N*)	Intervention description	Outcome measure(s)
Bagchi et al., 2011, USA	RCT	Spanish speaking	Improved communication between patient and provider	To evaluate whether availability of in-person professional interpreter services during ED visits affects satisfaction of limited English proficient patients and their health providers	ED: 2	In-person professional interpreter services in ED	Patient experience: patient understanding; patient satisfaction
					Intervention: 242		
					Control: 205		Staff experience: physician satisfaction; triage nurse satisfaction; discharge nurse satisfaction
Comstock et al., 2020, USA	RCT	African American	Improved patient self-management of health issue	To evaluate the efficacy and moderators of an educational intervention on Blood Pressure control	ED: 1	Education-based technology- mediated via kiosk, at discharge and at 7 weeks post-discharge, follow-up at 30, 90, and 180 days post-ED discharge. Three reminder phone calls	Patient outcomes: blood pressure related measures
					Intervention: 71		
					Control: 68		
Damluji et al., 2017, USA	Quasi-experimental	African American, Hispanic	Improved provider adherence to good clinical practice	To describe the clinical outcomes of implementation of standardised medical protocols for recognition and management of STEMI	ED: 15	STEMI care	System performance: EMS to ED time; ED elapsed time; ED to vascular access time; and door to balloon time
					Intervention: 5507		
Engel-Rebitzer et al., 2021, USA	RCT	Black, other — included American Indian, Asian, Pacific Islander, multiple races and other	Improved provider adherence to good clinical practice	To characterise racial disparities in opioid prescribing for acute pain and to test the hypothesis that racial disparities may be mitigated by giving clinicians additional information about their patients’ treatment preferences and risk of opioid misuse	ED: 4	Clinician-facing intervention consisting of a prepopulated form describing the patient’s treatment preference and risk of opioid misuse	Patient outcomes: pain score
					Intervention: 671		Patient experience: patients’ analgesia preferences vs prescribed analgesia
					Control: 341		
Field et al., 2009, USA	RCT	African American, Hispanic	Improved patient self-management of health issue	To evaluate potential ethnic differences in drinking outcomes following brief intervention in the trauma care setting	ED: 1	Brief motivational intervention including reinforcing self-efficacy and providing support for effort or intention to quit drinking to reduce associated harm	Patient outcomes: alcohol volume per week
					Intervention: 737		
					Control: 756		
Ford et al., 1997, USA	RCT	African American	Improved patient self-management of health issue	To assess the effects of an asthma education program on ED visits, limited days of activity, and asthma knowledge and beliefs for African American and Caucasian adults	ED: 2	Three small-group educational sessions on asthma, led by a trained healthcare professional	Patient outcomes: number of ED visits due to asthma; asthma knowledge and beliefs; number of days of limited activity due to asthma
					Intervention: 119		
					Control: 122		
Gany et al., 2007, USA	RCT	Spanish, Mandarin, or Cantonese-speaking	Improved communication between patient and provider	To evaluate the impact of interpreting method on patient satisfaction	ED: 1	Remote simultaneous medical interpreting	Patient experience: satisfaction with physician communication/care; satisfaction with interpreter
					Intervention: 371		
					Control: 364		
Garrick et al., 2019, USA	Case–control	Asian, Black, Hispanic/Latino, American Indian, Alaska Native, Pacific Islander, Middle Eastern	Improved health workforce capacity	To review the strategies implemented in the Highland Emergency Medicine Residency Program to increase the number of underrepresented minority emergency medicine residents	ED: 1	Highland emergency medicine residency program diversification initiative	System performance: number of underrepresented minorities graduated from emergency medicine residency
					Intervention: 174		
					Control: 174		
Jacobs et al., 2012, USA	Case–control	Spanish speakers	Improved communication between patient and provider	To measure the impact of a policy change from telephone and face-to-face interpreting to video-interpreting on ED care	ED: 2	Policy change from use of telephonic and face-to-face interpreting to use of a video-interpreting network	System performance: mean time in ED; mean number of laboratory tests, radiology services, electrocardiograms, and echocardiograms; rates of hospital admission for limited English proficient patients with chest and abdominal pain; percentage of patients leaving ED against medical advice
					Intervention: 9873		
					Control: 10, 928		
Kelso et al., 1995, USA	Cohort	African American	Improved patient self-management of health issue	To evaluate the impact of an ED asthma education initiative	ED: 1	One hour of asthma education in the ED before discharge	System performance: ED visits; hospital admissions
					Intervention: 30		Patient outcomes: medical treatments (patients using daily inhaled corticosteroids; patients using a spacer; patients using a home peak flow meter; patients on daily oral corticosteroids; patients taking theophylline; asthma knowledge (proper inhaler technique, knowledge of difference between inhaled corticosteroids and inhaled beta-agonists; trained in self-management)
					Control: 22		
					Intervention: 79		
					Control: 33		
Kwok et al. 2021, Canada	Qualitative	Mandarin, Cantonese, Punjabi, Russian, American Sign Language, Turkish	Improved communication between patient and provider	To improve communication between ED staff and patients who have limited understanding of spoken English	ED: 1	Interpreter on Wheels, Language insight audio and video interpreting service	System performance: virtual interpretation encounters (minutes)
					Intervention: 477		
McBride et al., 2016, Australia	Qualitative	Afghanistan, Sri Lanka, Iran	Improved health workforce capacity	To describe and evaluate the refugee health nurse liaison position	ED: 1	Refugee Health Nurse Liaisons supported staff and patients with a view of enhancing patient care, and building capacity within the sector to more effectively respond to the needs of this population group	System performance: access: number of interventions; number of referrals; number of education and capacity building sessions; narrative case studies
					Intervention: 946*		
Menchine et al., 2013, USA	RCT	Hispanic, Spanish language preference	Improved communication between patient and provider	To determine if verifying telephone numbers, obtaining best contact times, and informing patients that they will be contacted would increase the proportion of ED patients contacted at 48 to 72 h post-discharge	ED: 1	Research assistants verified telephone number, obtained best contact lines, and made four telephone attempts to contact each subject 48 to 72 h after discharge	System performance: successful telephone contact
					Intervention: 346		
					Control: 343		
Prendergast et al., 2021, USA	RCT	Black/African American, Hispanic/Latino, other	Improved patient self-management of health issue	To determine whether an ED education and empowerment intervention coupled with early risk assessment can help improve blood pressure in a high-risk population	ED: 1	Two intervention arms: 1) ED-initiated screening, brief intervention, and referral for treatment 2) Same as (1) + a 48–72 h post-acute care hypertension transition clinic	Patient outcomes: differences in mean systolic and diastolic blood pressure from baseline to study completion; proportion of participants with controlled hypertension at 9 months; change in hypertension knowledge score; change in medication adherence; limited bedside echocardiogram results
					Intervention: 99		
					Control: 51		
Oviedo Ramirez et al., 2018, USA	Cohort	Hispanic/mixed-origin	Improved patient self-management of health issue	To determine if the language in which brief intervention is delivered influences drinking outcomes among Mexican-origin young adults in the emergency department when controlling for ethnic matching	ED: 1	The Brief Negotiation Interview: engaging the patient and obtaining permission to discuss drinking, feedback, providing the patient with information about drinking norms, asking patient to discuss the pros and cons of their drinking, assessing the patient’s readiness to change their drinking, providing patient a list of options regarding making a change to their drinking, and negotiating with the patient their goal for reducing drinking and strategies for achieving this goal	Patient outcomes: no. of drinking days per week last 28 days; drinks per drinking day last 28 days; maximum drinks last 28 days; Short Inventory of Problems
					Intervention: 310		
					Control: NR		
Saban et al., 2019, Israel	Quasi-experimental	Arab, non-Jew	Improved provider adherence to good clinical practice	To evaluate whether a fast-track intervention program will reduce time-lags of patients with STEMI considering minority groups, various socioeconomic status and clinical risk factors	ED: 1	Fast track intervention for patients with chest pain — clinical guidelines adjusted, changes in electronic medical records (automatic notification and time exceeding alerts)	System performance: total time in ED; waiting time between ED exam and referral to cardiac catheterisation; door to balloon time; hospital LOS
					Intervention: 80		
					Control: 60		Patient outcomes: mortality

*ED* emergency department, *EMS* emergency medical service, *LOS* length of stay, *NR* not reported, *PTSD* post-traumatic stress disorder, *QoL* quality of life, *RCT* randomized controlled trial, *STEMI* ST-elevation myocardial infarction, *USA* United States of America

^*^Patients, 78% ED; 60 patient feedback survey

The interventions to improve healthcare for CALD patients were categorised into four strategies: (1) improving patient self-management of health issues (*n* = 6), (2) improving communication between patients and the health provider (*n* = 5), (3) improving adherence to good clinical practice (*n* = 3), and (4) building health workforce capacity (*n* = 2) (Table 2).

**Table 2:** Outcomes of ED interventions in Emergency Departments to improve healthcare for CALD adults

Strategy type	Author, year, country	Intervention	Outcome measure(s)	Control group		Intervention group	*P* value	Level of evidence^50^	Effect
Improved patient self-management of health issue	Ford et al., 1997, USA	**Asthma** Asthma education sessions	ED visits/year due to asthma– mean (SD)					II	
			Baseline	6.7 (8.4)		5.0 (3.6)			
			Post-intervention	4.8 (6.8)		2.7 (3.3)	0.93		ne
			Asthma knowledge and beliefs – mean (SD)						
			Baseline	14.3 (2.3)		14.1 (2.9)			
			Post-intervention	14.7 (2.3)		14.6 (3.2)	0.51		ne
			Number of days of limited activity due to asthma – mean (SD)						
			Baseline	27.8 (33.4)		20.6 (25.4)			
			Post-intervention	27.9 (55.7)		18.7 (36.8)	0.43		ne
Improved patient self-management of health issue	Kelso et al., 1995, USA	**Asthma** One hour of asthma education in the ED before discharge	ED visits – mean (SD)	3.3 (2.4)		3.5 (2.4)	0.67	III-2	ne
			Hospitalisations – mean (SD)	1.1 (1.2)		0.5 (0.8)	< 0.01*		+
Improved patient self-management of health issue	Comstock et al., 2020, USA	**Hypertension** Kiosk-based education intervention	Systolic blood pressure					II	
			Baseline – mean	159					
			180-day postintervention – mean	131		ns			ne
			Diastolic blood pressure						
			Baseline – mean	92					
			180-day postintervention – mean	84		ns			ne
Improved patient self-management of health issue	Prendergast et al., 2021, USA	**Hypertension** Two intervention arms: 1) ED-initiated screening, brief intervention, and referral for treatment 2) Same as 1) + a 48–72 hours post-acute care hypertension transition clinic	Change in systolic blood pressure from baseline to 6 months, mm Hg (race and hypertension treatment-adjusted) – mean (95% CI)	−17.6 (−23.9, −11.3)	−20.3 (−26.7, −13.9)	−20.6 (−27.1, −14.1)	0.04*	II	+
			Change in systolic blood pressure from baseline to 9 months, mm Hg (race and hypertension treatment-adjusted) – mean (95% CI)	−19.9 (−25.9, −13.8)	−25.6 (−31.7, −19.4)	−23.6 (−29.9, −17.4)	0.09		ne
			Change in diastolic blood pressure from baseline to 6 months, mm Hg (race and hypertension treatment-adjusted) – mean (95% CI)	−8.6 (−11.6, −4.9)	−10.2 (−13.6, −6.8)	−8.6 (−12.0, −5.1)	0.18		ne
			Change in diastolic blood pressure from baseline to 9 months, mm Hg (race and hypertension treatment-adjusted) – mean (95% CI)	−8.9 (−12.4, −5.4)	−11.9 (−15.1, −7.8)	−11.4 (−15.1, −7.8)	0.06		ne
			Controlled hypertension at 6 months (race and hypertension treatment-adjusted) – % (95% CI)	18.2 (7.5, 28.9)		18.9 (11.2, 26.6)	0.61		ne
			Controlled hypertension at 9 months (race and hypertension treatment-adjusted) – % (95% CI)	23.2 (11.7, 34.8)		29.5 (20.5, 38.4)	0.38		ne
Improved patient self-management of health issue	Field et al., 2009, USA	**Substance Misuse +/- Mental Health** Brief motivational intervention	Effects of brief motivational intervention on alcohol volume per week – X^2^ (df)			3.0 (2)	0.22	II	ne
Improved patient self-management of health issue	Oviedo Ramirez et al., 2018, USA	**Substance Misuse +/- Mental Health** The Brief Negotiation Interview	Number of drinking days per week at 3-month follow-up – β (SE)			0.99 (0.06)	0.32	III-2	ne
			Number of drinking days per week at 12-month follow-up – β (SE)			0.14 (0.08)	0.89		ne
			Number of drinks per drinking day at 3-month follow-up – β (SE)			0.08 (0.05)	0.94		ne
			Number of drinks per drinking day at 12-month follow-up – β (SE)			-0.45 (0.05)	0.66		ne
			Maximum drinks in a day at 3-month follow-up – β (SE)			-0.22 (0.06)	0.83		ne
			Maximum drinks in a day at 12-month follow-up – β (SE)			−0.05 (0.06)	1.0		ne
			SIPs + 6 count last 3 months at 3-month follow-up – β (SE)			0.57 (0.51)	0.60		ne
			SIPs + 6 count last 3 months at 12-month follow-up – β (SE)			−1.10 (0.33)	0.27		ne
Improved communication between patient and provider	Bagchi et al., 2011, USA	**Interpreter services** Professional interpreter services	Patient understanding – *n* (%); OR (95%CI)	191 (17.8)		231 (93.0)	0.01*	II	+
				61.2 (22.5, 65.7)					
			Patient satisfaction – *n* (%); OR (95%CI)	190 (23.9)		231 (95.8)	0.01*		+
				71.9 (30.8, 167.4)					
			Physician satisfaction – *n* (%); OR (95%CI)	192 (17.1)		228 (94.8)	0.01*		+
				87.9 (39.0, 197.9)					
			Triage nurse satisfaction – n (%); OR (95%CI)	227 (94.3)		198 (22.3)	0.01*		+
			Discharge nurse satisfaction – *n* (%); OR (95%CI)	180 (17.8)		188 (94.6)	0.01*		+
				81.2 (63.6, 103.6)					
Improved communication between patient and provider	Gany et al., 2007, USA	**Interpreter services** Remote simultaneous medical interpreting	Satisfaction with Physician Communication/Care – mean (SD), β (95% CI)	0.5 (0.3)		0.5 (0.4)	ns	II	
			Satisfaction with Interpreter – mean (SD), β (95% CI)	0.5 (0.4)		0.5 (0.4) 0.07 (−0.01, 0.15)	ns		
Improved communication between patient and provider	Jacobs et al., 2012, USA	**Interpreter services** Policy change from use of telephonic and face-to-face interpreting to use of a video-interpreting network	Hospital A					III-2	
			Time in ED (minutes) – mean	585		697	ns		ne
			Laboratory tests – mean	31.5		29.1	ns		ne
			Radiology tests – mean	2.5		2.2	ns		ne
			EKG – mean	2.9		2.6	ns		ne
			Echocardiograms – mean	0.3		0.2	ns		ne
			Admissions – *n* (%)	1,681 (44)		954 (39)	ns		ne
			Left against medical advice – *n* (%)	239 (6.0)		88 (3.6)	0.04*		+
			Hospital B						
			Time in ED (minutes) – mean	532		573	ns		ne
			Laboratory tests – mean	2.9		2.8	ns		ne
			Radiology tests – mean	0.6		0.6	ns		ne
			EKG – mean	0.3		0.2	ns		ne
			Echocardiograms – mean	0		0	ns		ne
			Admissions – *n* (%)	476 (17)		110 (16)	ns		ne
			Left against medical advice – *n* (%)	298 (11)		57 (8)	ns		ne
Improved communication between patient and provider	Kwok et al. 2021, Canada	**Interpreter services** Interpreter on Wheels	Virtual interpretation encounters – *n* (minutes)				477 (4,123)	-	-
Improved communication between patient and provider	Menchine et al., 2013, USA	**Follow-up contact** Verifying telephone numbers, obtaining best contact times, and informing patients that they will be contacted	Successful telephone contact					II	
			Hispanic – OR (95% CI)				3.55 (1.73, 7.29)*	-	
			Black/African American – OR (95% CI)				1.57 (0.67, 3.68)	-	
			Other race – OR (95% CI)				1.11 (0.37, 3.05)	-	
Improved provider adherence to good clinical practice	Engel-Rebitzer et al., 2021, USA	**Opioid Prescribing** Prepopulated form describing the patient’s treatment preference and risk of opioid misuse	Received an opioid prescription					II	
			Black patients preferring opioids – OR (95% CI)			0.8 (0.3, 2.6)	ns		ne
			Other race patients preferring opioids – OR (95% CI)			0.7 (0.2, 3.0)	ns		ne
			Black patients preferring no opioids – OR (95% CI)			0.3 (0.0, 0.5)	ns		ne
			Other race patients preferring no opioids – OR (95% CI)			0.5 (0.1, 2.3)	ns		ne
Improved provider adherence to good clinical practice	Saban et al., 2019, Israel	**STEMI management** Fast track intervention for patients with chest pain	Total time in ED – mean (SD)	72.0 (56.4)		37.4 (19.6)	< 0.01*	III-1	+
			Waiting time between ED exam and referral to cardiac catherisation – mean (SD)	37.9 (26.5)		42.5 (30.7)	0.36		ne
			Door to Balloon Time – mean (SD)	106.3 (60.5)		79.9 (38.1)	< 0.01*		+
			Hospital LOS – mean (SD)	5.4 (3.2)		5.9 (3.2)	0.73		ne
			Mortality – *n* (%)	8 (13.3)		6 (7.5)	0.25		ne
Improved provider adherence to good clinical practice	Damluji et al., 2017, USA	**STEMI management** Reducing time to reperfusion	EMS to ED time (minutes) – median	37		31	< 0.01*	III-1	+
			ED elapsed time (minutes) – median	30		16	< 0.01*		+
			ED to vascular access time (minutes) – median	50		34	< 0.01*		+
			Door to balloon time (minutes) – median	68		49	< 0.01*		+
Improved health workforce capacity	Garrick et al., 2019, USA	Highland Emergency Medicine Residency Program Diversification Initiative	Underrepresented minorities in graduating classes – % (95% CI)	12.1		27.1	–	III-2	+
						(6.0, 24.1)			
Improved health workforce capacity	McBride et al., 2016, Australia	Refugee Health Nurse Liaison – to build capacity within the health sector to respond effectively to the needs of asylum seekers and refugees	Referred patients who received a comprehensive assessment – *n* (%)			937 (99)	-	-	+
			RHNL helpful throughout their hospital visit – %			77	-		+
			Having someone who respected and understood their culture was the most valued aspect of their interaction with the RHNL – %			90	-		+
			RHNL ensured patients understood what was happening to them – %			86.6	-		+
			RHNL assisted with accessing interpreting services – %			63.3	-		+
			RHNL provided helpful information – %			24	-		+

*ASI* Addiction Severity Index (score range, 0–1), *CALD* culturally and linguistically diverse, *ED* emergency department, *EKG* electrocardiogram, *EMS* emergency medical services, GAD generalized anxiety disorder (score range, 0–21), *HSCL* Hopkins Symptom Checklist (score range, 0–4), *IIDEA* Integrated Intervention for Dual Problems and Early Action, *ne* no effect, *LSSS* Liverpool Seizure Severity Scale, *MADRS* Montgomery-Asberg Depression Rating Scale, *MCS* Mental Component Score, *NHE* negative health event, *ns* not significant, *OR* odds ratio, *PCL* Posttraumatic Stress Disorder Checklist (score range, 0–80), *PCS* Physical Component Score, *PHQ* Patient Health Questionnaire (score range, 0–27), *QOLIE-10* Quality of Life in Epilepsy, *RHNL* Refugee Health Nurse Liaison, *SD* standard deviation, *SF-36* Short-Form 36, *SIPS* + *6* Short Inventory of Problems, *SMART* self-management for people with epilepsy and a history of negative health events intervention, + positive effect, − negative effect

^*^Statistically significant outcome

### Patient Self-management of Health Issues

#### Asthma

Two studies [32, 33] provided education for asthma self-management and measured patient and system performance outcomes (Table 1). Ford et al. [32] provided three education sessions to African American and non-African American participants, collecting socio-demographic factors to compare groups (Table 2). While the authors stated the African American intervention cohort were less likely to have a post-high school education or an income above US$15,000 and answered fewer asthma knowledge and beliefs questions correctly pre-intervention, it is unclear whether the education was then tailored to the African American cohort. The authors report 49% of the African American cohort and 25% of the non-African American cohort dropped out of formal education. Post-intervention, there was little change in knowledge and beliefs; the African American group had a significantly lower mean asthma knowledge and beliefs score than the non-African American group. The authors report a non-significant reduction in asthma-affected activity and ED visits.

Kelso et al. [33] provided a 1-hour intensive education session in the ED to African American individuals as they presented. While socio-demographic data was not collected, education was individualised comprising demonstration and observation of use of inhalers, spacers, education about medications, colour-coded peak flow measurement and interpretation, identification of triggers, and self-management techniques. All participants demonstrated increased knowledge. ED visits and hospitalisations reduced significantly compared to the control group.

#### Hypertension

Two studies [34, 35] provided education for hypertension self-management and measured patient outcomes (Table 1). Comstock et al. [34] provided kiosk-based education in the ED for an African American cohort with uncontrolled hypertension. Socio-economic factors showed only 48.2% of the intervention group were employed, 63% had health insurance, and 87% did not have a high school diploma. Notably, 85.6% were reported to have adequate health literacy. It is unknown whether these factors impacted the education provided, but patients that could not use the education kiosk were excluded from the study (Supplement 3). Most participants (52.5%) did not reach the 180-day follow-up. Those who did achieved a significant reduction in systolic blood pressure (159 to 131 mmHg), but there was no significant difference between groups. More participants with higher education and more without diabetes achieved BP control, but not to significance.

While Prendergast et al. [35] cited racial/ethnic disparities in hypertension control, their study recruited all individuals with uncontrolled hypertension (systolic > 160 mmHg) presenting to the ED who were fluent in either English or Spanish. Participants were randomised across enhanced usual care, ED-based screening and intervention, and multicomponent intervention groups. Baseline knowledge and ethnicity was collected across groups, but it is unclear if this influenced the education provided. No significant impact on knowledge or mean blood pressure (BP) were found (Table 2); however, 29.5% of participants in the intervention group and 23.2% of the enhanced usual care group achieved BP control (systolic < 140 mmHg) at 9 months.

#### Substance Misuse

Two studies [36, 37] implemented intervention to influence patients reduced use of substances and reported patient outcomes (Table 1). Field et al. [36] described significant differences in drinking characteristics for ethnic minorities, more negative health and social consequences, and complex treatment needs often compounded by socio-economic factors. Their intervention targeted English or Spanish speakers from African American or Hispanic groups and a ‘White’ group, all of whom presented injured to the ED. Their brief motivational intervention reinforcing self-efficacy and providing support for effort or intention to quit drinking to reduce associated harm excluded persons in police custody in the ED and those without an identifiable residence. Factors were reported to compare participants across treatment and intervention groups and as potential confounders; both African American and Hispanic cohorts were less likely to be employed, less likely to have post-high school education, and earned less. The Hispanic cohort self-reported a decrease in weekly consumption of 9.1 standard drinks; however, non-significant reductions were reported in heavy drinking across all groups (Table 2).

Oviedo Ramirez et al. [37] similarly delivered a brief intervention in the ED but targeted a Hispanic/mixed-origin population due to their known high level drinking behaviour. Participants randomised to the intervention were able to select the preferred language of delivery (English or Spanish). Whereas 86.8% of those choosing English were born in the USA, only 56.7% of those choosing Spanish were born in the USA. Hispanic/mixed-origin community health workers were recruited to deliver the intervention in the individuals’ language of choice. Patients in the intervention showed significant reductions in maximum number of drinks per occasion at 3 months and in all outcomes of alcohol consumption at 12 months. There were no significant differences between language groups (Table 2).

## Communication Between Patients and the Health Provider

Five studies sought to improve communication between patients and the health providers in the ED. Bagchi et al. [38] and Gany et al. [39], who measured patient experience, as well as Jacobs et al. [40] and Kwok et al. [41], who measured system performance, all implemented an intervention using interpreter services. Menchine et al. [42] implemented an intervention to improve follow-up of patients discharged from ED, measuring system performance (Table 1).

Bagchi et al. [38] compared standard interpreter services (telephone interpretation and ad hoc provided by bilingual staff and family/friends of the patient) with a professionally trained medical interpreter on site for Spanish speaking-limited English proficient patients. Staff and patients were predominantly very satisfied with the in situ interpreter, showing a significant difference to the control group (Table 2). Gany et al. [39] trialled remote medically trained simultaneous (speaking at the same time as they are hearing content) interpreters, in-person consecutive (speaking in language blocks) interpreters, for Spanish, Mandarin, or Cantonese-speaking limited English language groups, and a non-interpreter group who were comfortable speaking English. There was no significant difference in staff or patient satisfaction in communication across the groups (Table 2), although patients using remote interpretation felt their privacy was better protected. All patient groups reported poor satisfaction with feeling understood by physicians, explanations of procedures, results, and discharge instructions.

Jacobs et al. [40] reviewed the implementation of a video-interpreter service that increased access to interpreters within a minute of the request by comparing a Spanish-speaking group and English-speakers across two hospital sites (Table 1). Compared with prior telephone and face-to-face interpreters, there was no difference in the number of tests performed, ED length of stay, or admissions across two sites. However, the risk of patients leaving the ED before receiving appropriate care was significantly reduced in the Spanish-speaking cohort (Table 2).

Kwok et al. [41] implemented a video interpreter during the COVID-19 pandemic to reduce the time bilingual staff were used ad hoc in this role. The mode was used 477 times in 2 months, for Mandarin, Cantonese, Punjabi, Russian, Turkish, and American Sign Language.

Menchine et al. [42] introduced a short intervention for Hispanic people who preferred Spanish language to collect patient contact details prior to discharge to improve telephone follow-up. Most intervention participants (78.1%) were from one CALD background, had less than high school education (42.4%), an income < US$15,000, and no health insurance (51.3%). Twenty (5.9%) participants lived in a shelter or were homeless, and 104 of all intervention participants (25.8%) did not own a mobile phone. There was no significant difference in contact between usual care and intervention.

## Provider Adherence to Good Clinical Practice

Three studies [43–45] implemented interventions to improve clinical practice, measuring patient outcomes and/or system performance. One study sought improvement in known disparities for opioid prescribing. Two studies sought whole of population improvement for acute chest pain or ST-elevation myocardial infarction (STEMI) management, where CALD groups were reported as sub-analyses.

### Opioid Prescribing

Engel-Rebitzer et al. [44] describe a study undertaken in four EDs to reduce disparity in opioid prescribing and included ‘Black’, ‘White’, and ‘Other’ (self-identified as American Indian, Asian, Pacific Islander and those who reported multiple races or other race) participants. On admission to the ED, participants undertook a validated survey to assess their risk of opioid misuse and a pain management preference survey. Those who could not use the technology platform or were without internet access or a smartphone were excluded from the study. In the experimental arms, this information was provided to ED clinicians treating the individual. Despite the information, ‘Black’ patients who reported a preference for opioids were less likely than similar ‘White’ patients to be discharged with an opioid prescription. ‘White’ patients who did not prefer opioids were more likely than similar ‘Black’ patients to be discharged with an opioid prescription (Table 2).

### Chest Pain and ST-Elevation Myocardial Infarction (STEMI) Care

Damluji et al. [43] describe an intervention to improve rapid recognition of STEMI and early treatment in a culturally diverse community with indicators of low socio-economic status; 53% had no post-high school education, and 34.5% were below the US poverty threshold. Commitment to management protocols was incorporated in emergency medical services (EMS) and 15 EDs, measuring time to treatment variables (Table 2). Retrospective analysis of the pre-intervention cohort revealed treatment disparities in quality metrics of STEMI care among subpopulations. Coordination of EMS and hospital-based systems of care through the development of a standardised protocol was associated with an overall improvement in door-to-balloon time (DTBT) and ED treatment time metrics (Table 2) and contributed to reversal of disparities across subpopulations of STEMI care.

Saban et al. [45] implemented an intervention to fast-track patients presenting to the ED with chest pain in a population where Arab and non-Jew groups experienced lower socio-economic status. Despite the intervention, the CALD patients still experienced prolonged time to electrocardiogram and DTBT in comparison with the non-CALD group (Table 2).

## Health Workforce Capacity

Two studies [46, 47] describe interventions aimed at building health workforce capacity among ED staff to care for CALD communities, measuring patient satisfaction and/or system performance. Garrick et al. [46] describe a strategy to widen access to ED physician training to achieve more diversity in the workforce. The program developed revised recruitment processes to weigh alternative applicant qualities, affirmatively increasing the diversity profile of the intake over a 10- to 20-year period.

McBride et al. [47] describe the work of three ED-based refugee health nurse liaisons (RHNL) in a community with a high number of asylum seekers and refugees, measuring system performance and patient outcomes. Comprehensive assessment of patients presenting to ED was undertaken to link in appropriate services and to build cultural competency among the staff. Over a 12-month pilot, 937 patients were referred to the RHNL, 80% of whom required RHNL support to address identified health and social issues, with 58% requiring ≥ 1 referral. Patients valued the RHNL service, respect for their culture, and having someone ensure they knew what was happening to them (Table 2).

## Discussion

**Fig. 2:**
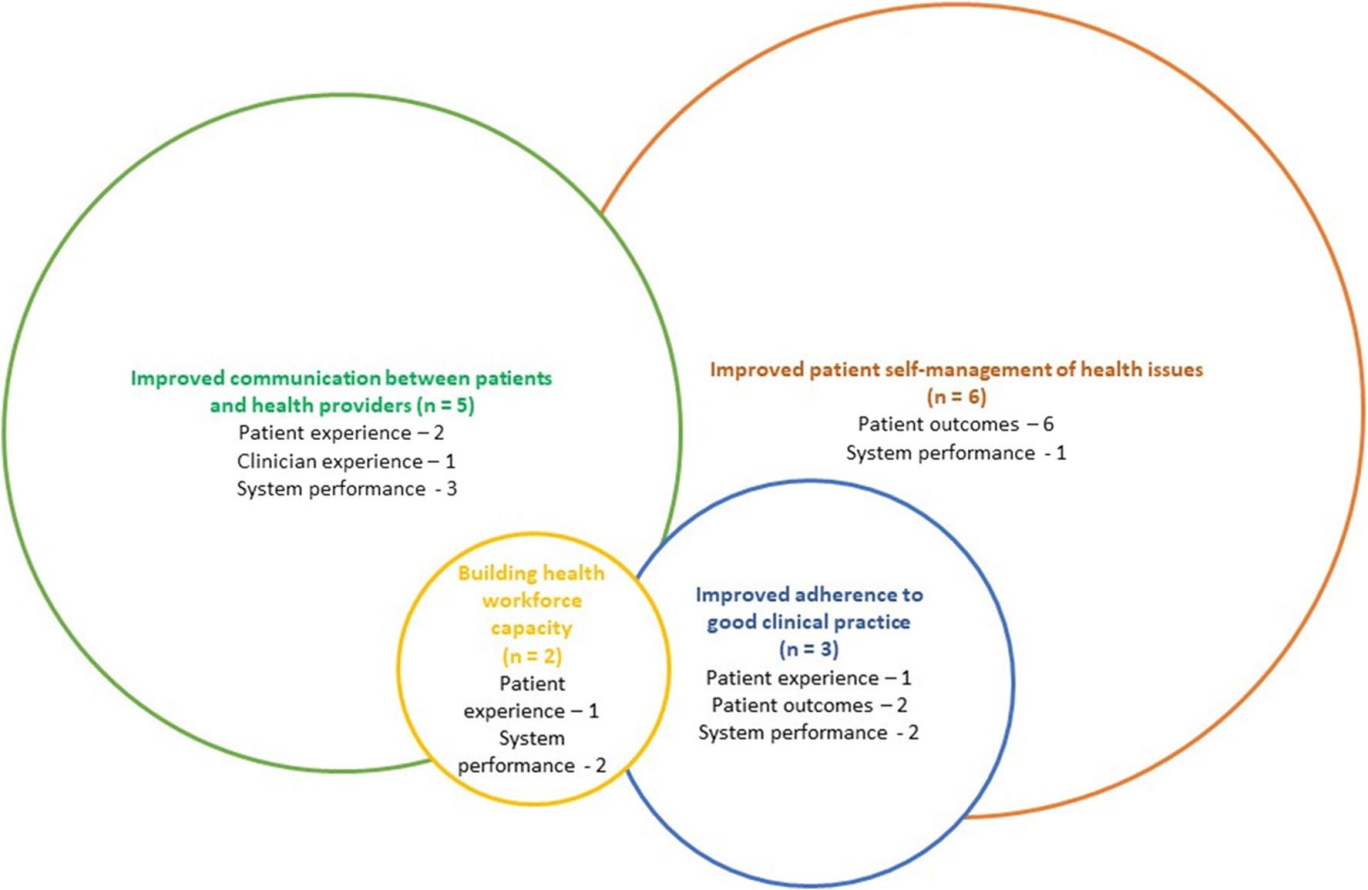
Strategies to improve delivery of care in emergency departments for CALD communities and reported positive effect on patient or clinician satisfaction, system performance, or patient outcomes

Emergency departments are an important gateway into the health system for people from CALD communities. In contrast to the growing literature describing health disparity for people from a CALD background, this review demonstrates the dearth of reported ED interventions to improve healthcare delivery for CALD adults. Nevertheless, we have identified four clear strategies that may inspire further action: (i) improved patient self-management, (ii) improved communication between patients and health providers, (iii) improved adherence to good clinical practice, and iv) improved health workforce capacity (Fig. 2). This review also highlighted important considerations for interventions including careful definition of the CALD group, intervention design that considers the socio-economic features of the population of interest, and more active examination of institutional structures that limit access. Measures of only one construct — patient experience, clinician experience, system performance, or patient outcomes — may not always reveal the value of quality improvement initiatives.

There is international inconsistency with the definition of CALD groups. Country of origin, such as ‘Mexican-origin’, and terms, such as ‘Black’, ‘White’, ‘Asian’, and ‘Caucasian’, were also problematic, with no further information to elucidate which populations are defined under each term. Broad definitions, such as CALD that are based on culture, religion or language minorities, or a combination of any of these, assist in avoiding racialisation based on phenotypic characteristics [31], a trait that has been linked to racial stratification, ideology, and differential access to resources [20]. Three communication-based studies utilised lower English proficiency (LEP) and Spanish language as determinants of ethnic minority status. While this determining feature is sensible for an interpreter-based study, English proficiency is not the primary indicator of ethnic minority status, as ethnic minority groups are heterogeneous and characterised by other social and demographic inequities. Initiatives targeting LEP are significant to the overall aim in dismantling service gaps; however, they do not take into consideration groups that are impacted by disparities but speak English. One of the starkest examples is the marked disparity in healthcare experienced by African Americans, who communicate in English.

Socio-economic factors were considered by some, but not all, studies, and no studies considered religion or sexual orientation. Some interventions assumed a financial or technological minimum such as having a stable address, a smart phone, or computer literacy, with no consideration of those who did meet these requirements. Others illuminated differences in knowledge attainment between CALD and non-CALD groups without addressing the level of literacy required for the intervention despite there being existing disparity at baseline. Interventions would benefit from consideration of the intersecting socio-economic inequities of the population of interest at the design stage so as not to diminish self-esteem among already stigmatised minority groups, and to promote inclusivity. An alternative approach was demonstrated by Kelso et al. [33] where an individualised, learner-oriented intervention was delivered in the ED to all persons in the population of interest and appeared successful in impacting better self-management of asthma. Similarly, Oviedo Ramirez et al. [37] recruited community health workers from the same CALD group of interest and prepared them to deliver the intervention, which was provided in the participants’ language of preference.

Institutional racism focuses on the outcomes that result from default practices and norms [4]. Factors that contribute to racism in healthcare include lack of diversity, poor healthcare quality, poor clinical communication, barriers to access, and poor uptake [20]. The challenge is that institutional racism in health care is rarely visible to those that are privileged by it [4, 20]. Two studies in this review [44, 45] illuminate disparity in health care for two CALD groups perpetrated by existing health practice. In addition, Damluji et al. [43] highlight the incidental discovery of disparity for people from a CALD background, reversed through commitment to excellent practice for all. These examples justify continued scrutiny of health outcomes for CALD communities, coupled with interventions tailored to the population of interest. Needham et al. [20] provide some guidance for researchers including consideration of whether to take a minority health approach, in which people who identify as a specific CALD group are the focus of the analysis, or to compare differences in exposure to, or effect of, policies for different CALD and non-CALD groups*.* Definition of CALD status needs to be routinely collected consistently by healthcare services to accurately define populations and measure outcomes, but this may be challenging if the population perceives disclosure may lead to unfair treatment. The importance of strategies to build health workforce diversity and capacity are also apparent in this context. Widening access strategies in health professional education premise that more socio-culturally appropriate care will be delivered by a health workforce that is representative of the diversity of the population [48].

Measures of intervention success and contextual factors also warrant consideration. Unvalidated satisfaction surveys are challenging to judge [49]. Older age, lower education, and lower income level have all been indicated as predictors of higher patient satisfaction [50]. With so many EDs unable to move admitted patients to hospital beds in a timely manner [51], the impact of an intervention on ED length of stay may also be confounded. In the papers reviewed, interventions targeting self-management of health issues showed improved patient outcomes (self-reported and ED re-presentation data) and system performance (reduced hospitalisations). System performance measures were clear metrics for clinical pathways that aimed to streamline care for high acuity patients. Careful selection of metrics [49] and attention to known confounders will assist in more clearly elucidating the impact of ED interventions for adults from a CALD background.

## Limitations

To our knowledge, this is the first review of interventions to improve ED care for CALD adults. This research was limited to interventions that primarily targeted people from a CALD background. This definition varies between countries; some include Indigenous populations while others do not. To respect this position, we included papers from countries that included Indigenous populations as a CALD group, but excluded papers reporting intervention specifically for Indigenous populations from countries such as Australia where they are not considered a CALD group. This review also excluded interventions that targeted children, which led to the exclusion of studies conducted in paediatric EDs. More information on the interaction of CALD parents in the ED setting, especially the effect of communication-based interventions, would have been useful. This review included only articles published in the literature which excluded relevant, but unpublished material. Only interventions published in the English language were included in this study. Studies in languages other than English are likely to be valuable in this area.

## Conclusions

Given reported and latent health disparities, scrutiny of health outcomes for CALD communities coupled with tailored interventions addressing identified inequity is warranted. Strategies could include improving self-management of health issues, improving communication between patients and health providers, improving adherence to good clinical practice, and building health workforce capacity. Measuring the impact on health system performance and patient outcomes relies on consistent and accurate collection of CALD status in health service data and careful selection of quality metrics.

## Data Availability

Provided.
